# Mental health symptoms and discrimination among immigrant and US-born Hispanic or Latino adults during the COVID-19 pandemic

**DOI:** 10.1186/s40359-025-02798-7

**Published:** 2025-05-13

**Authors:** Emmanuel A. Odame, Maryam Elhabashy, David Adzrago, Jolyna Chiangong, Cameron K. Ormiston, Faustine Williams

**Affiliations:** 1 Department of Environmental Health Sciences, School of Public Health, University of Alabama at Birmingham, Birmingham, AL USA; 2 Division of Intramural Research, National Institute on Minority Health and Health Disparities, 11545 Rockville Pike, Rockville, MD 20852 USA; 3 Icahn School of Medicine at Mount Sinai, New York, NY USA

**Keywords:** COVID-19, Hispanic or Latino, Mental health, Depression, Anxiety, Loneliness, Discrimination, Immigrant health

## Abstract

**Background:**

Mental health symptoms are highly stigmatized, potentially increasing the risk of experiencing discrimination, particularly within minoritized communities such as Hispanic or Latino populations. Thus, stigmatizing views of mental health symptoms may exacerbate exposure to experiencing discrimination, including xenophobia. The Hispanic or Latino population has increasingly been vulnerable to mental health symptoms since the COVID-19 pandemic and has historically faced persistent discrimination. However, few studies have explored associations between mental health symptoms and discrimination, especially when accounting for and stratifying by birthplace. This study estimates the prevalence of discrimination by mental health symptoms among Hispanic or Latino individuals in the United States (US). It also examines the associations between mental health symptoms and discrimination, stratified by birthplace.

**Methods:**

A national online cross-sectional survey was distributed between May 13, 2021, and January 9, 2022, among US adults (*N* = 5,413). The analytical sample included Hispanic or Latino individuals (*n* = 1,126) who were US-born (*n* = 625) and foreign-born (*n* = 501). Chi-square tests were used to assess bivariate differences in everyday discrimination (measured with the Everyday Discrimination Scale). Polytomous logistic regressions were conducted to examine everyday discrimination and its correlates, adjusting for sociodemographic factors.

**Results:**

A higher proportion of Hispanic or Latino individuals reported experiencing discrimination daily or weekly (38.19%) compared to monthly (16.25%). Discrimination was more frequently reported among US-born individuals as well as those with anxiety, depression, anxiety/depression, and a higher level of loneliness. Foreign-born individuals were significantly less likely to experience discrimination compared to their US-born counterparts. Anxiety/depression and loneliness were associated with higher risks of experiencing discrimination. In analyses stratified by birthplace, loneliness was a significant risk factor for discrimination among both groups. Anxiety/depression was a significant risk factor only among US-born individuals.

**Conclusions:**

Mental health symptoms and birthplace were significantly associated with experiences of discrimination among Hispanic or Latino individuals, with more pronounced risks for US-born individuals. These findings highlight birthplace-related disparities and broader disparities in mental health and discrimination within the growing US Hispanic or Latino population. More research should explore potential mechanisms for bidirectional associations between mental health symptoms and discrimination among minoritized communities.

**Supplementary Information:**

The online version contains supplementary material available at 10.1186/s40359-025-02798-7.

## Background

Discrimination—unfairly or prejudicially treating people and groups on grounds of their characteristics, including but not limited to immigration status, nationality, race, gender, age, or sexual orientation—is a major public health issue [1–4]. Discrimination has been associated with a variety of physiological effects, including increased heart rate, heightened blood pressure, and elevated cortisol levels [2, 5–7]. Those who endure discrimination often exhibit stronger negative emotional reactions, which can lead to unhealthy behaviors such as substance abuse, poor sleep, and reduced physical activity [1–6, 8]. As a result, repeated exposure to discrimination, whether by stigmatizing in-group individuals or prejudiced out-group individuals, has been connected to numerous health risks, including cardiovascular disease and increased mortality rates [1–4, 9]. A robust body of research has examined discrimination as a risk factor for various negative physical and mental health outcomes [1–8]. However, current literature is limited by largely unidirectional observations of the relationship between discrimination and mental health, examining discrimination as a predictor for mental health symptoms, despite evidence that mental health symptoms may in fact be a predictor for experiencing discrimination, especially among racially and/or ethnically minoritized populations [6, 10]. A systematic review and meta-analysis indicate that individuals with mental health problems experience stigmatization and discrimination [11]. Similarly, the American Psychiatric Association also reported that people with mental health problems are marginalized and discriminated against [12].

Given the negative perceptions of mental health symptoms across racially and/or ethnically minoritized communities, the burdens of discrimination (e.g., xenophobia), personal shame, and public stigma can disproportionately impact individuals believed to be experiencing mental illness [12–15]. One California study found that, regardless of race and/or ethnicity, participants mostly believed that individuals with mental health problems, including loneliness, anxiety, depression, or psychological distress, encountered high levels of prejudice and discrimination [14]. The same study also indicated that a substantial proportion of participants reported being discriminated against due to their mental illness. In addition to mental health, research suggests that perceptions of discrimination may vary by birthplace [16]. Mixed evidence indicates that discrimination can vary in form and internalization, with foreign-born individuals (i.e., immigrants) being more likely to experience institutional or systemic discrimination that may become more recognizable as length of stay increases, compared to minoritized US-born individuals who have been found to report higher rates of interpersonal everyday discrimination [17, 18].

Among the Hispanic or Latino populations, past research has suggested that US-born individuals experience higher rates of mental health symptoms than immigrants/foreign-born individuals [19]. However, immigrants, particularly racial and/or ethnic minority groups, experience high burdens of discrimination due to factors such as lower English-language proficiency, immigration status (e.g., naturalized citizens, non-citizens, refugees, permanent residents, unauthorized immigrants), and country of origin [17, 18, 20–23]. The rising anti-immigrant sentiments further compound the unfair treatment and experiences of immigrants [17, 21, 22]. Despite evidence that mental health and birthplace may be considerable predictors for experiencing discrimination, there is a lack of studies investigating the potential nuances within ethnically and/or racially minoritized populations.

The Mental Illness Stigma Framework posits that mental illness stigma, including societal stigma of mental illness, leads to discrimination [24]. According to this framework, people with mental health illness are subjected to four common forms of discrimination: withholding help, avoidance, segregation, and coercion [24]. Minority stress theory, for instance, indicates that minority individuals have poorer socioeconomic conditions and/or multiple disadvantaged identities that increase their risks for stress, discrimination, and poor health outcomes [25–30]. Together, these theoretical frameworks suggest that people, including Hispanic or Latino individuals, with multiple minority identities may face compounded risk of experiencing discrimination. Past research has also documented self-stigma as a prevalent construct among Hispanic or Latino individuals experiencing mental health symptoms [31–35]. This self-stigma may lower self-esteem and empowerment, and incur a deeper sense of being disadvantaged or minoritized [31–35]. Hispanic or Latino individuals account for 44% of the US immigrant population and are known to face numerous disparities, putting them at disproportionally heightened risk for poor mental health and discrimination [36–40].

Past studies estimate that US-born minoritized individuals generally report higher rates of mental health symptoms and experiences of discrimination [19, 41–43]. However, more evidence suggests that the mental health of Hispanic or Latino immigrant populations (vs. non-immigrants) has been more severely impacted in recent years, due to their disproportionate representation in frontline and/or essential work, and heightened risk of exposure to COVID-19 [44–48]. A study found that 24% of Americans believe that immigrants burden the country by taking jobs, housing, and health care [37], a sentiment that can influence discriminatory treatment based on place of birth. For immigrant persons, the current surge in discrimination and anti-immigrant sentiments is harmful to their general well-being [21, 22, 49]. Immigrant individuals often undergo an acculturative period during which they attempt to adapt to the culture of the host country [49, 50]. During this transitory period, some immigrants experience discrimination due to linguistic differences, diet, and cultural norms [49, 51]. Yet, to date, no study has examined mental health symptoms and the associated risks of reported discriminatory incidents based on birthplace among Hispanic or Latino individuals during the COVID-19 pandemic.

To address these gaps, this current study aims to (1) examine whether birthplace (US-born vs. foreign-born) and mental health symptoms are associated with discrimination, and (2) assess whether these associations vary by birthplace among Hispanic or Latino adults, while both adjusting, and not adjusting for sociodemographic factors. Based on empirical studies, Mental Illness Stigma Framework, minority stress theory, and self-stigma [25–27, 31, 35], we hypothesized that among Hispanic or Latino individuals, mental health symptoms would be positively associated with reports of discrimination. We also hypothesized that the association between mental health symptoms and reports of discrimination would be weaker among foreign-born individuals compared to US-born individuals. This second hypothesis was informed by recent research on mental health and discrimination by nativity as well as the healthy immigrant paradox, which suggests that foreign-born individuals report better health than native-born counterparts in their host countries [41–43, 52, 53]. This study, which is exploratory in nature, also seeks to aid in hypothesis generation for future research. In this context, the prevalence of discrimination and the perceived reasons for discrimination were also assessed. By utilizing disaggregated data, this study also provides a more comprehensive understanding of Hispanic or Latino population health and encourages more holistic and tailored approaches to scientific inquiry and intervention development.

## Methods

The study was a national online cross-sectional survey conducted between May 13, 2021, and January 9, 2022, among US adults aged 18 years and older. Racially and/or ethnically minoritized, low-income (< $25,000 annual household income), and rural adults were oversampled to ensure adequate representation of these target subpopulations. Qualtrics LLC was contracted to facilitate recruitment for the web-based survey. They distributed the survey to a national sample of racial and/or ethnic groups based on American Community Survey population estimates. The survey was administered through proprietary consumer panels and pre-arranged pools of respondents who consented to participate in surveys by market research services via social media and/or email. The 116-item survey assessed the participants’ mental health, discrimination, and sociodemographic characteristics during the COVID-19 pandemic. Further information regarding the survey design and implementation has been reported elsewhere [54]. Of the 10,000 surveys distributed to the participants, 5,938 were returned to the research support firm, Information Management Services, Inc., who after expert review and fraud detection deemed 5,413 surveys (91.6%) as valid and usable for analysis. Our current analysis was restricted to the participants who identified their race/ethnicity as Hispanic or Latino with complete responses (*n* = 1,126). The Institutional Review Board (IRB) at the National Institutes of Health (NIH) approved the study on December 23, 2020 (IRB #000308). The study was performed in accordance with the ethical standards as laid down in the 1964 Declaration of Helsinki and its later amendments or comparable ethical standards. Informed consent was obtained from all individuals who participated in this study.

### Measures

The dependent variable for this study was self-reported discrimination frequency during the COVID-19 pandemic. It was determined based on an adapted version of the 5-item Everyday Discrimination Scale, and categorical representation of discrimination frequency was utilized in accordance with the scale’s recommendations [55, 56]. This scale has been validated in the US and used frequently to examine discrimination during the pandemic [55, 57, 58]. Participants were asked how often any of the following things or items happened to them (response options: 5 = daily or almost daily, 4 = 2 to 3 times a week, 3 = about once a week, 2 = about once a month, 1 = never [reference category]) since the beginning of the COVID-19 pandemic. The items include: (1) You are treated with less courtesy or respect than other people; (2) You receive worse service than other people in restaurants or stores; (3) People act as though they think you are not intelligent; (4) People act as though they are afraid of you; and (5) You are threatened or assaulted. Due to small samples, which could result in skewed distributions, for the response options and within the covariate categories, we collapsed the responses to determine discrimination frequency as experienced daily or weekly (about once a week, 2–3 times a week, daily or almost daily), monthly (about once a month), or never experienced discrimination. This categorization will accommodate variable distribution and reduce skewness.

To report perceived reasons for discrimination, participants were asked, “What do you think is the main reason for these discrimination experiences? Check all that apply.” Respondents selected from a list of twelve options: people think I have coronavirus/COVID-19, race, ancestry or national origin, immigration status, gender, age, religion, height, weight, sexual orientation, education or income level, or none of these or not applicable.

Independent variables for this study included place of birth and mental health symptoms (anxiety, depression, anxiety/depression, and loneliness), which were examined as potential risk factors for discrimination frequency. Place of birth involved whether the participants were US-born/non-immigrant if they were born in the US, or foreign-born/immigrant if they were born outside the US.

Loneliness was assessed separately from anxiety and depression, as it can uniquely and differentially impact the risk of developing depressive and/or anxiety symptoms [59]. Additionally, loneliness may have played a significant role in shaping perceptions of discrimination during the COVID-19 pandemic [60, 61]. The mental health symptoms were measured using the following validated instruments: 2-item Generalized Anxiety Disorder scale (GAD-2) for anxiety symptoms, with total scores of 0–6 [62, 63]; 2-item Patient Health Questionnaire (PHQ-2) for depressive symptoms, with total scores of 0–6 [62, 63]; 4-item Patient Health Questionnaire (PHQ-4) for anxiety/depressive symptoms, with total scores of 0–12 [62, 63]; and 3-item UCLA Loneliness scale for loneliness (short version; scores ranged from 3 to 9, with higher scores indicating higher loneliness) [64, 65]. The reliability of both the PHQ-4 and UCLA Loneliness scale has been well-established, with PHQ-4 scale demonstrating sufficient internal consistency for anxiety/depression (α = 0.92), anxiety (α = 0.90), and depression (α = 0.86), and the 3-item UCLA Loneliness scale demonstrating sufficient internal consistency for loneliness (α = 0.72) [66–69]. Dichotomous variables were created in accordance with the recommendations of the respective instruments (i.e., GAD-2 scores *≥* 3 indicate presence of anxiety symptoms, PHQ-2 scores *≥* 3 indicate presence of depressive symptoms, and PHQ-4 scores of *≥* 3 indicate presence of anxiety/depressive symptoms) to determine the presence or absence of the symptoms [62, 63]. That is, these cutoffs allow for identification of clinically significant symptoms. Because anxiety and depressive symptoms are co-occurring or frequently comorbid symptoms [62, 63], we assessed their individual and combined effects to determine their unique associations with discrimination.

The covariates or confounding factors included in the analyses were the following sociodemographic factors: Hispanic or Latino subgroups (Mexican, Mexican American, Chicano; Puerto Rican; Cuban/Cuban American; Dominican Republic; Central American; South American; Another Hispanic, Latino, or Spanish origin), age, gender identity (man, woman, or something else/non-binary/transgender), sexual orientation (heterosexual, lesbian/gay, bisexual, or something else), and marital status (single/never married, divorced/widowed/separated, or married). We also included level of education completed (less than high school, high school graduate/GED, some college/technical school, and *≥* college degree), annual household income (less than $25,000, $25,000 to < $35,000, $35,000 to < $50,000, $50,000 to < $75,000, and *≥ *$75,000), employment status (employed or unemployed), and homelessness/unstable housing (yes or no).

### Statistical analyses

Descriptive and bivariate statistics of discrimination frequency were computed across the sociodemographic factors and the mental health symptoms. We first estimated the prevalence of discrimination frequency. Second, we evaluated the prevalence of discrimination frequency across the mental health symptoms, birthplace and the covariates (sociodemographic factors). One-Way ANOVA was used to compare the mean scores of loneliness by the discrimination frequency categories. Chi-square (*χ*^2^) tests were used to compare the prevalence of discrimination frequency across the categorical variables such as the mental health symptoms (anxiety status, depression status, and anxiety/depression status) and sociodemographic factors (birthplace, Hispanic or Latino subgroups, age, gender identity, sexual orientation, marital status, level of education completed, annual household income, employment status, and homelessness/unstable housing). Next, we assessed the reported reasons for perceived discrimination frequency before stratifying the reasons by place of birth. The level of statistical significance was determined at *p* < 0.05.

Polytomous logistic regression models were used to examine the associations of place of birth and mental health symptoms with discrimination frequency (Reference category = never: daily/weekly vs. never and monthly vs. never), adjusting for the sociodemographic factors. Additionally, we stratified the association between mental health symptoms and discrimination frequency by place of birth, adjusting for the sociodemographic factors. We assessed multicollinearity among the independent variables included in the models and found no significant collinearity (variance inflation factor was less than 10). We estimated and reported relative risk ratios (RRRs) with 95% confidence intervals (CIs) for the polytomous logistic regression models. We performed all statistical analyses with Stata version 16.1.

## Results

### Sociodemographic and mental health characteristics of the overall sample

Table 1 summarizes the sociodemographic characteristics and mental health symptoms within the overall sample (*n* = 1,126). More than half of the overall sample experienced anxiety/depressive symptoms (53.46%), while close to a third experienced anxiety (31.35%) or depressive (31.44%) symptoms. The mean loneliness score was 5.07 (SD = 2.00), with total scale scores ranging from 3 to 9. The participants were generally US-born/non-immigrant persons (55.51%), Mexicans, Mexican Americans, or Chicanos (45.83%), aged 35–49 years (28.95%), identified their gender as a woman (61.63%) or sexual orientation as heterosexual (85.52%), married (52.75%), had completed at least a college education (35.08%), had less than $25,000 annual household income (24.96%), were employed (56.75%), and were not homeless (86.59%).

**Table 1:** Descriptive and bivariate statistics of discrimination frequency across mental health symptoms and sociodemographic factors among Hispanics or Latinos (*n* = 1,126)

	Discrimination frequency				
	Total sample	Daily or at least once a week	About once a month	Never	
	*n* (%)	*n* (%)	*n* (%)	*n* (%)	*P*-value
**Overall**		430 (38.19)	183 (16.25)	513 (45.56)	
**Mental health symptoms**					
**Anxiety symptoms**					< 0.001
No	773 (68.65)	208 (26.91)	137 (17.72)	428 (55.37)	
Yes	353 (31.35)	222 (62.89)	46 (13.03)	85 (24.08)	
**Depressive symptoms**					< 0.001
No	772 (68.56)	206 (26.68)	141 (18.26)	425 (55.05)	
Yes	354 (31.44)	224 (63.28)	42 (11.86)	88 (24.86)	
**Anxiety/Depressive symptoms**					< 0.001
No	524 (46.54)	98 (18.70)	86 (16.41)	340 (64.89)	
Yes	602 (53.46)	332 (55.15)	97 (16.11)	173 (28.74)	
Loneliness score (Mean: SD)	1,126 (5.07: 2.00)	430 (5.92: 1.91)	183 (5.31: 1.96)	513 (4.27: 1.78)	< 0.001
**Place of birth**					< 0.001
Foreign-born (Immigrant)	501 (44.49)	160 (31.94)	76 (15.17)	265 (52.89)	
US-born (Non-immigrant)	625 (55.51)	270 (43.20)	107 (17.12)	248 (39.68)	
**Hispanic or Latino subgroups**					0.380
Mexican, Mexican American, Chicano	516 (45.83)	211 (40.89)	82 (15.89)	223 (43.22)	
Puerto Rican	178 (15.81)	66 (37.08)	30 (16.85)	82 (46.07)	
Cuban/Cuban American	79 (7.02)	24 (30.38)	9 (11.39)	46 (58.23)	
Dominican Republic	63 (5.60)	25 (39.68)	10 (15.87)	28 (44.44)	
Central American	57 (5.06)	19 (33.33)	11 (19.30)	27 (47.37)	
South American	137 (12.17)	44 (32.12)	22 (16.06)	71 (51.82)	
Another Hispanic, Latino, or Spanish origin	96 (8.53)	41 (42.71)	19 (19.79)	36 (37.50)	
**Age**					< 0.001
18–25 years	249 (22.11)	122 (49.00)	42 (16.87)	85 (34.14)	
26–34 years	279 (24.78)	129 (46.24)	49 (17.56)	101 (36.20)	
35–49 years	326 (28.95)	124 (38.04)	59 (18.10)	143 (43.87)	
50–64 years	177 (15.72)	44 (24.86)	24 (13.56)	109 (61.58)	
*≥ *65 years	95 (8.44)	11 (11.58)	9 (9.47)	75 (78.95)	
**Gender identity**					0.012
Man	402 (35.70)	171 (42.54)	58 (14.43)	173 (43.03)	
Something else/ non-binary/transgender	30 (2.66)	17 (56.67)	6 (20.00)	7 (23.33)	
Woman	694 (61.63)	242 (34.87)	119 (17.15)	333 (47.98)	
**Sexual orientation**					0.001
Heterosexual	963 (85.52)	350 (36.34)	149 (15.47)	464 (48.18)	
Lesbian/Gay	56 (4.97)	27 (48.21)	11 (19.64)	18 (32.14)	
Bisexual	87 (7.73)	46 (52.87)	16 (18.39)	25 (28.74)	
Something else	20 (1.78)	7 (35.00)	7 (35.00)	6 (30.00)	
**Marital Status**					0.432
Single/Never married	408 (36.23)	158 (38.73)	73 (17.89)	177 (43.38)	
Married	594 (52.75)	225 (37.88)	86 (14.48)	283 (47.64)	
Divorced/Separated/Widowed	124 (11.01)	47 (37.90)	24 (19.35)	53 (42.74)	
**Education**					0.040
Less than high school	97 (8.61)	50 (51.55)	11 (11.34)	36 (37.11)	
High school graduate or GED	284 (25.22)	119 (41.90)	43 (15.14)	122 (42.96)	
Some college or technical school	350 (31.08)	124 (35.43)	56 (16.00)	170 (48.57)	
*≥ *College degree	395 (35.08)	137 (34.68)	73 (18.48)	185 (46.84)	
**Annual household income**					0.045
Less than $25,000	281 (24.96)	108 (38.43)	39 (13.88)	134 (47.69)	
$25,000 to < $35,000	200 (17.76)	85 (42.50)	36 (18.00)	79 (39.50)	
$35,000 to < $50,000	182 (16.16)	80 (43.96)	31 (17.03)	71 (39.01)	
$50,000 to < $75,000	227 (20.16)	88 (38.77)	35 (15.42)	104 (45.81)	
$75,000 or more	236 (20.96)	69 (29.24)	42 (17.80)	125 (52.97)	
**Employment status**					< 0.001
Employed	639 (56.75)	277 (43.35)	102 (15.96)	260 (40.69)	
Unemployed	487 (43.25)	153 (31.42)	81 (16.63)	253 (51.95)	
**Homelessness/Unstable housing**					< 0.001
No	975 (86.59)	317 (32.51)	171 (17.54)	487 (49.95)	
Yes	151 (13.41)	113 (74.83)	12 (7.95)	26 (17.22)	

### Bivariate associations of and main reasons for discrimination with sociodemographic and mental health measures

A higher proportion of participants reported experiencing discrimination daily or weekly (38.19%) compared to those who experienced discrimination monthly (16.25%) (Table 1). There were statistically significant differences in discrimination frequency based on place of birth, age, gender identity, sexual orientation, level of education completed, annual household income, employment status, homelessness/unstable housing, and mental health symptoms. A higher proportion of those who experienced discrimination daily or at least once a week were US-born/non-immigrant persons (43.20%), aged 18–25 years (49.00%), identified their gender as something else/non-binary/transgender (56.67%) or sexual orientation as bisexual (52.87%), had completed less than high school education (51.55%), more than $35,000 to < $50,000 annual household income (43.96%), were employed (43.35%), or experienced homelessness/unstable housing (74.83%). These higher proportions were also observed among those with anxiety (62.89%), depression (63.28%), anxiety/depression (55.15%), or had a mean loneliness score of 5.92 (SD = 1.91).

Those who experienced discrimination about once a month were mostly US-born/non-immigrant persons (17.12%), aged 35–49 years (18.10%), identified their gender as something else/non-binary/transgender (20.00%) or their sexual orientation as something else (35.00%), had completed at least a college education (18.48%), more than $25,000 to < $35,000 annual household income (18.00%), were unemployed (16.63%), or experienced no homelessness/unstable housing (17.54%). These higher proportions were also observed among those who experienced no anxiety (17.72%), no depression (18.26%), no anxiety/depression (16.41%), or had a mean loneliness score of 5.31 (SD = 1.96).

**Fig. 1:**
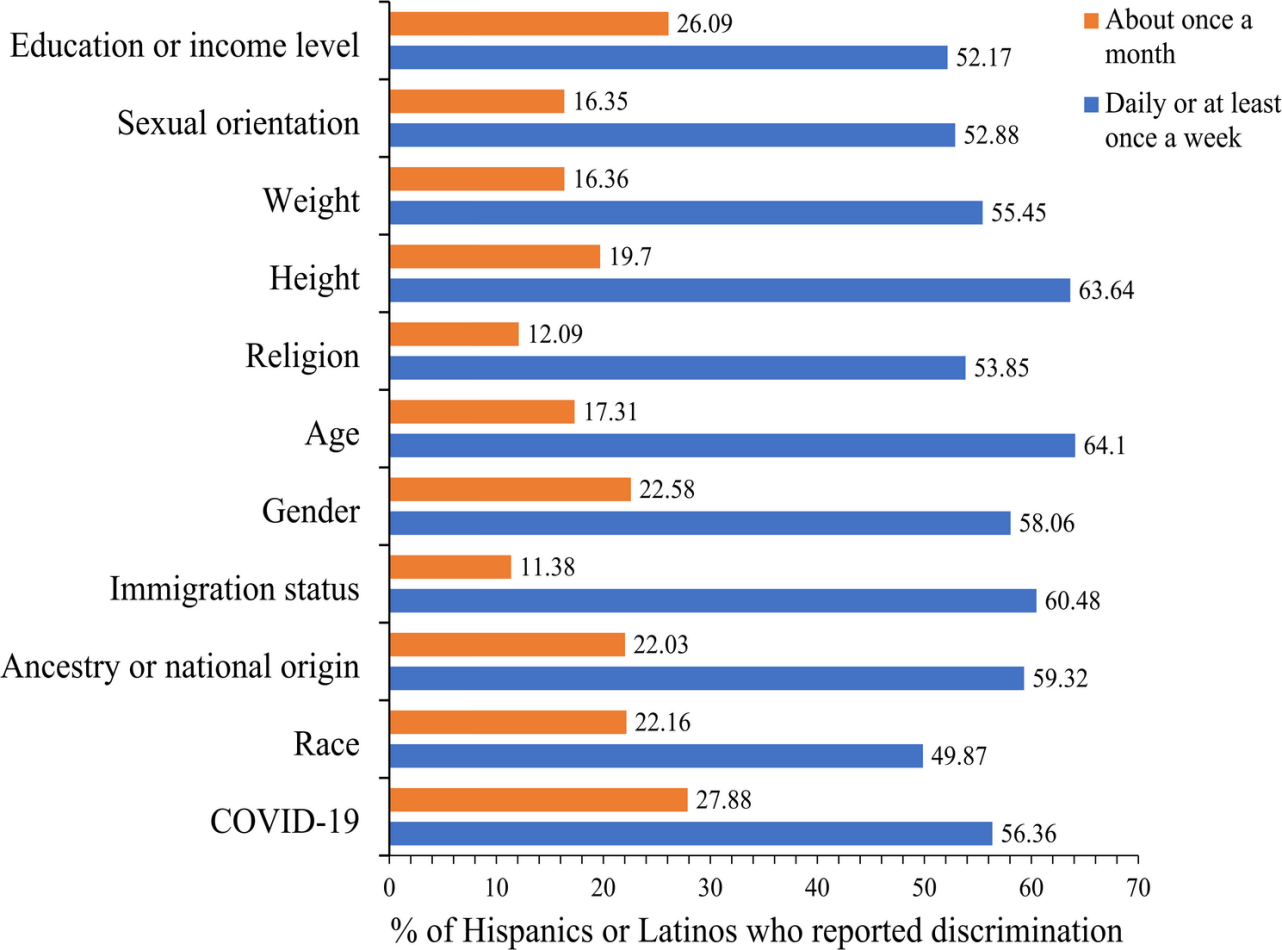
Reasons for discrimination frequency among Hispanics or Latinos in the United States who reported experiencing discrimination. *Reasons provided were not mutually exclusive

Figure 1 presents the main reported reasons (the reasons provided were not mutually exclusive) for discrimination against the participants. More than half of the participants reported education/income level, sexual orientation, weight or height, religion, age, gender, immigration status, ancestry/national origin, or COVID-19 status as the main reasons for discrimination daily or at least once a week.

**Fig. 2:**
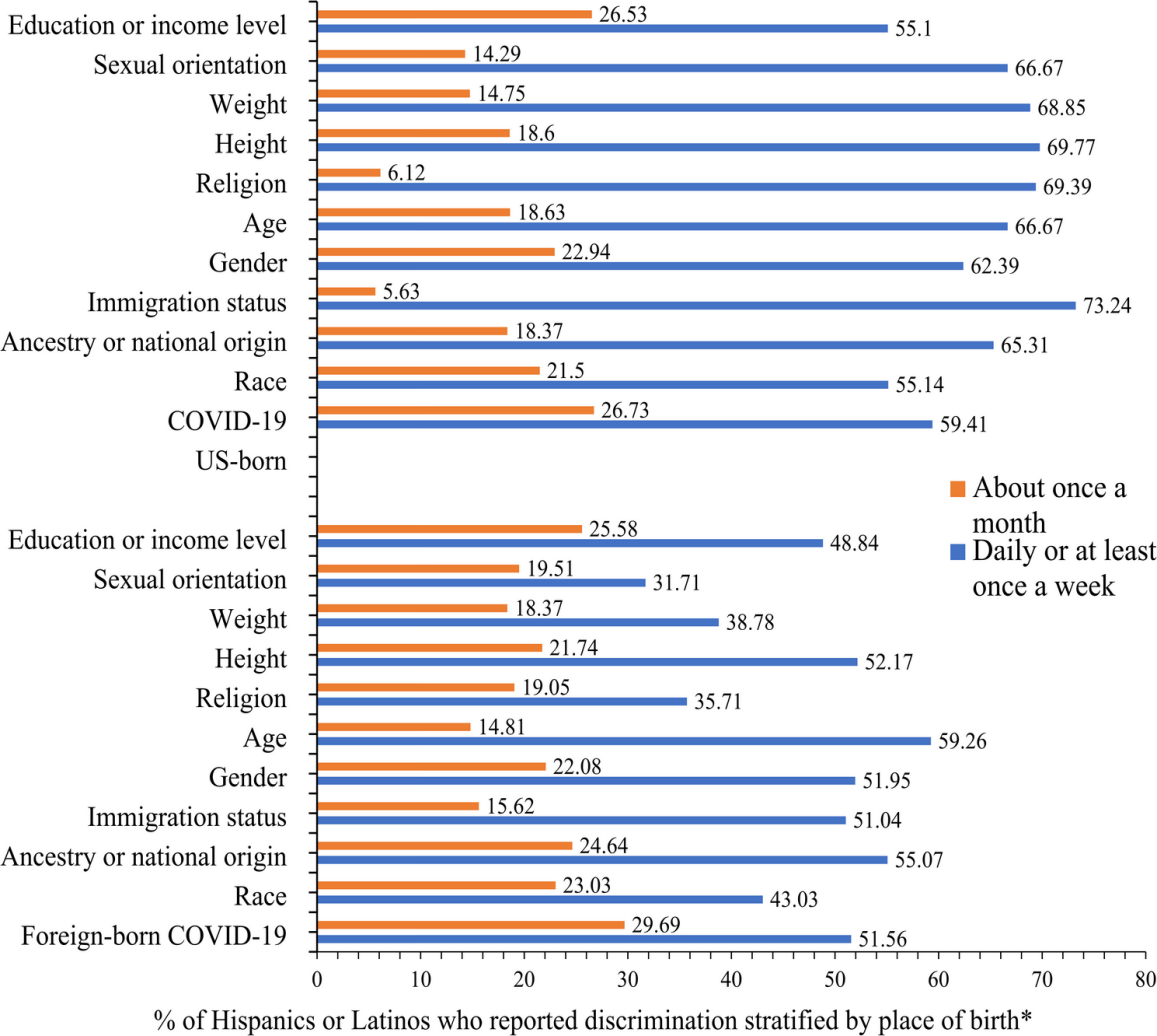
Reasons for discrimination frequency stratified by place of birth among Hispanics or Latinos in the United States who reported experiencing discrimination. *Reasons provided were not mutually exclusive

The main reasons were further stratified by the participants’ place of birth (Fig. 2). Overall, the main reasons, which are not mutually exclusive, were reported among a higher proportion of US-born/non-immigrant persons than foreign-born/immigrant persons, especially in the case of discrimination daily or at least once a week.

### Place of birth and mental health factors associated with discrimination

Table 2 presents the polytomous logistic regression models that examined three-level discrimination frequency (daily or at least once a week vs. never and about once a month vs. never) and its associated place of birth and mental health factors, adjusting for the sociodemographic factors. Compared to US-born/non-immigrant individuals, foreign-born/immigrant individuals were 30% less likely to experience daily/weekly discrimination (95% CI = 0.50, 0.96), and 34% less likely to experience monthly discrimination (95% CI = 0.45, 0.95). Presence of anxiety/depressive symptoms increased risk for daily/weekly discrimination by 138% (95% CI = 1.54, 3.70), and for monthly discrimination by 68% (95% CI = 1.03, 2.75). Higher loneliness scores were also significantly associated with higher risk for discrimination, with each unit increase in loneliness incurring 29% higher risk for daily/weekly discrimination (95% CI = 1.17, 1.42) and 33% higher risk for monthly discrimination (95% CI = 1.19, 1.49). Discrimination frequency was not significantly associated with anxiety and depression as separate factors. In additional polytomous logistic regression models (eTable 1.1 supplement 1), where anxiety/depression was included in a separate model due to high collinearity and all mental health factors analyzed as continuous variables, loneliness scores were associated with a higher risk of discrimination. Depression scores were associated with an increased risk of daily/weekly discrimination, whereas anxiety scores were not associated with discrimination. In a separate model that excluded anxiety and depression (eTable 1.2 supplement 1), loneliness scores remained significantly associated with a higher risk of discrimination, while anxiety/depression scores were significantly associated with a higher risk of daily/weekly discrimination. Immigrants (vs. non-immigrants) continued to show higher risks for discrimination in these additional models.

**Table 2:** Polytomous logistic regression analysis of the associations between mental health symptoms and place of birth related with reports of discrimination frequency among Hispanics or Latinos (*n* = 1,126)

	Discrimination frequency (Reference Category: Never)			
	Daily or at least once a week		About once a month	
	RRR	95% CI	RRR	95% CI
**Mental health symptoms**				
**Anxiety**				
No	Ref		Ref	
Yes	1.53	(0.96, 2.45)	0.84	(0.47, 1.51)
**Depression**				
No	Ref		Ref	
Yes	1.26	(0.79, 2.02)	0.56	(0.31, 1.01)
**Anxiety/Depression**				
No	Ref		Ref	
Yes	2.38***	(1.54, 3.70)	1.68*	(1.03, 2.75)
Loneliness score	1.29***	(1.17, 1.42)	1.33***	(1.19, 1.49)
**Place of birth**				
Foreign-born (Immigrant)	0.70*	(0.50, 0.96)	0.66*	(0.45, 0.95)
US-born (Non-immigrant)	Ref		Ref	
**Age**				
18–25 years	Ref		Ref	
26–34 years	0.73	(0.46, 1.18)	0.90	(0.52, 1.58)
35–49 years	0.63	(0.39, 1.01)	0.81	(0.47, 1.42)
50–64 years	0.33***	(0.19, 0.58)	0.38**	(0.19, 0.75)
*≥*65 years	0.22***	(0.10, 0.50)	0.26**	(0.10, 0.64)
**Gender identity**				
Man	Ref		Ref	
Something else/non-binary/transgender	0.79	(0.25, 2.45)	0.79	(0.21, 2.95)
Woman	0.44***	(0.31, 0.63)	0.86	(0.57, 1.28)
**Sexual orientation**				
Heterosexual	Ref		Ref	
Lesbian/Gay	1.62	(0.77, 3.42)	2.02	(0.88, 4.62)
Bisexual	1.42	(0.77, 2.61)	1.40	(0.68, 2.87)
Something else	1.25	(0.34, 4.61)	5.13*	(1.39, 18.96)
**Marital status**				
Single/Never married	Ref		Ref	
Married	1.76**	(1.20, 2.57)	1.17	(0.75, 1.80)
Divorced/Separated/Widowed	2.19*	(1.20, 3.97)	2.24*	(1.16, 4.33)
**Education**				
Less than high school	Ref		Ref	
High school graduate or GED	0.91	(0.50, 1.68)	1.40	(0.62, 3.14)
Some college or technical school	0.76	(0.41, 1.39)	1.32	(0.59, 2.94)
*≥*College degree	0.93	(0.49, 1.74)	1.88	(0.83, 4.28)
**Annual household income**				
Less than $25,000	Ref		Ref	
$25,000 to < $35,000	1.61	(0.99, 2.61)	1.73	(0.97, 3.08)
$35,000 to < $50,000	1.55	(0.92, 2.60)	1.65	(0.89, 3.07)
$50,000 to < $75,000	1.58	(0.95, 2.65)	1.48	(0.81, 2.74)
$75,000 or more	1.12	(0.64, 1.94)	1.79	(0.96, 3.36)
**Employment status**				
Employed	Ref		Ref	
Unemployed	0.63**	(0.44, 0.89)	1.01	(0.67, 1.52)
**Homelessness/Unstable housing**				
No	Ref		Ref	
Yes	4.38***	(2.60, 7.39)	1.10	(0.52, 2.30)

RRR = Relative risk ratio. 95% CI = 95% confidence interval. Statistical significance at *p < 0.05, **p < 0.01, and ***p < 0.001. Ref = reference

The models were further stratified by place of birth (Table 3). Among foreign-born/immigrant participants, loneliness was the only significant factor associated with discrimination frequency. Each unit increase in loneliness heightened risk for daily/weekly discrimination by 29% (95% CI = 1.12, 1.48) and for monthly discrimination by 36% (95% CI = 1.15, 1.61). Among US-born/non-immigrant participants, experiencing anxiety/depressive symptoms increased risk of daily/weekly discrimination by 269% (95% CI = 1.98, 6.85) and for monthly discrimination by 139% (95% CI = 1.23, 4.62). In a supplementary model (eTable 2.1 supplement 1), again, anxiety/depression was analyzed separately due to high collinearity. In this model, loneliness scores were associated with an increased risk of discrimination among both foreign-born and US-born individuals. Depression scores were associated with a higher risk of daily/weekly discrimination among US-born individuals. In another separate supplemental model that excluded both anxiety and depression (eTable 2.2 supplement 1), loneliness scores were associated with a higher risk for discrimination irrespective of birthplace. In this model, anxiety/depression scores were associated with a higher risk of daily/weekly discrimination among both foreign-born and US-born Hispanic or Latino individuals.

**Table 3:** Polytomous logistic regression analysis of the associations between mental health symptoms and reports of discrimination among foreign-born and US-born Hispanics or Latinos, adjusting for sociodemographic characteristics

	Foreign-born (Immigrant)		US-born (Non-immigrant)	
	Reference Category: Never		Reference Category: Never	
	Daily or at least once a week	About once a month	Daily or at least once a week	About once a month
	**RRR (95% CI)**	**RRR (95% CI)**	**RRR (95% CI)**	**RRR (95% CI)**
**Mental health symptoms**				
**Anxiety**				
No	Ref	Ref	Ref	Ref
Yes	1.74 (0.84, 3.60)	1.71 (0.67, 4.36)	1.35 (0.71, 2.56)	0.50 (0.23, 1.12)
**Depression**				
No	Ref	Ref	Ref	Ref
Yes	1.26 (0.61, 2.63)	0.40 (0.16, 1.03)	1.31 (0.70, 2.48)	0.65 (0.29, 1.42)
**Anxiety/Depression**				
No	Ref	Ref	Ref	Ref
Yes	1.55 (0.80, 2.97)	1.19 (0.55, 2.58)	3.69*** (1.98, 6.85)	2.39* (1.23, 4.62)
Loneliness score	1.29** (1.12, 1.48)	1.36*** (1.15, 1.61)	1.29*** (1.12, 1.48)	1.36*** (1.17, 1.59)

RRR = Relative risk ratio. 95% CI = 95% confidence interval. Statistical significance at *p < 0.05, **p < 0.01, and ***p < 0.001. Ref = reference

Table 2 also displays covariates which were included in this study. Compared to individuals aged 18–25 years, individuals aged 50–64 years had 67% lower risks of experiencing daily/weekly discrimination (RRR = 0.33, 95% CI = 0.19, 0.58), and 62% lower risks of experiencing monthly discrimination (RRR = 0.38, 95% CI = 0.19, 0.75). Similarly, individuals aged at least 65 years had 78% lower risks of experiencing daily/weekly discrimination (RRR = 0.22, 95% CI = 0.10, 0.50), and 74% lower risks of experiencing monthly discrimination (RRR = 0.26, 95% CI = 0.10, 0.64). Women had a 56% lower risk of experiencing daily/weekly discrimination (RRR = 0.44, 95% CI = 0.31, 0.63) compared to men. Unemployed individuals also had a 37% lower risk of experiencing daily/weekly discrimination (RRR = 0.63, 95% CI = 0.44, 0.89) compared to employed individuals.

Compared to heterosexual individuals, a 413% higher risk of monthly discrimination was observed among individuals who identified their sexual orientation as something else (RRR = 5.13, 95% CI = 1.39, 18.96). Individuals who experienced homelessness/unstable housing were 338% more likely to experience daily/weekly (RRR = 4.38, 95% CI = 2.60, 7.39) compared to those who did not experience homelessness/unstable housing. Being married increased risk of daily/weekly discrimination by 76% (RRR = 1.76, 95% CI = 1.20, 2.57), and being divorced/separated/widowed increased risk of daily/weekly discrimination by 119% (RRR = 2.19, 95% CI = 1.20, 3.97), compared to being single/never married. Among divorced/separated/widowed individuals, this risk also extended to monthly experiences of discrimination, with 124% higher risk among these individuals (RRR = 2.24, 95% CI = 1.16, 4.33).

## Discussion

The Hispanic or Latino population is the largest, youngest, and one of the fastest growing minority groups in the US [70, 71]. Despite the size and growth of this population, this is the first study to examine associations of mental health symptoms and place of birth with discrimination in the US Hispanic or Latino population based on place of birth. Evaluating mental health- and discrimination-related disparities within this understudied population, especially during the pandemic, is essential to informing more tailored interventions and resource allocation for addressing mental health symptoms to mitigate discrimination and improve population health.

US-born Hispanic or Latino participants in this study were more likely than their foreign-born counterparts to report discrimination if they experienced mental health symptoms. They also reported higher frequencies of daily/weekly discrimination. This may be partly explained by the fact that non-immigrant Latinos have been found to exhibit higher risks of anxiety and depressive symptoms compared with their immigrant counterparts [19]. Additionally, non-immigrants in general have been found to report higher rates of discrimination than immigrant individuals [16, 41, 72]. Relatedly, the ethnic resilience perspective suggests that increased exposure to the host society can lead to heightened ethnic consciousness and awareness of inequality and negative stereotypes, ultimately leading to greater attributions of discriminatory treatment to ethnic identity over time rather than other minoritized identities [72–74]. If foreign-born Hispanic or Latino individuals possess a less acute understanding and/or internalization of microaggression and prejudiced behavior, this may help explain the weaker associations between anxiety/depression and discrimination within this subgroup. However, more studies are needed to evaluate how acculturation influences perceptions of discriminatory experiences among foreign-born Hispanic or Latino individuals, as well as the potential manifestations and impacts of in-group discrimination due to mental health stigma. This can aid in the development of interventions to target subgroups such as US-born individuals who may be more vulnerable to discrimination due to experiencing various mental health symptoms.

While our findings supported the hypotheses that mental health symptoms would be associated with discrimination across subgroups, with stronger associations among US-born individuals, there are nuances that should be elucidated. Firstly, our findings indicated that within the Hispanic or Latino population, individuals experiencing loneliness were more likely to experience discrimination, regardless of birthplace. This pervasive impact of loneliness on discrimination relates to past studies, which have found loneliness as a uniquely potent risk factor for negative psychological outcomes [75–77] and a significant contributor to perceived experiences of discrimination [78]. Neuroendocrine, neural, and behavioral responses to loneliness can increase alertness to potential harm from other individuals and alter impulse control [79, 80], which may increase an individual’s internalization of interpersonal interactions as discriminatory. Furthermore, since Hispanic or Latino culture is generally more collectivistic than individualistic, feelings of loneliness due to separation and/or other barriers to social support [77] may further contribute to elevated risks of discrimination. These findings on loneliness contribute to growing bodies of work investigating the possible cyclical relationship between loneliness and discrimination [81, 82], the role of social support in perceptions of discriminatory experiences [77, 78], and the role of (un)impaired cognitive function on perceiving and internalizing discrimination [83, 84]. However, further exploration in these areas is needed, especially in relation to other symptoms, such as anxiety and/or depression. Better understandings of these dynamics could elucidate potential risk factors and interventions for minority communities, such as the Hispanic or Latino population.

Anxiety/depression symptoms also influenced discrimination among US-born Hispanic or Latino individuals, potentially explaining the higher risk of perceived discrimination within this subgroup compared to foreign-born counterparts. This additional risk among US-born individuals may be due to stigmatizing perceptions of mental health symptoms within the Hispanic or Latino population [85, 86]. However, past literature suggests that stigma toward mental health may vary by disorder type, and cultural beliefs may be stronger predictors of stigma than birthplace [87, 88]. Therefore, more research should explore inter-racial and intra-racial experiences of stigma, particularly by investigating culture-specific and illness-specific variations that may differ by birthplace. While the survey used in this study did not include questions regarding whether discrimination was from in-group or out-group individuals, future research should explore the potential exposure to in-group vs. out-group discrimination among minority individuals experiencing mental health symptoms.

It should be noted that while daily/weekly discrimination was mostly reported by individuals with anxiety, depression, or anxiety/depression, those who experienced discrimination less frequently (i.e., monthly, or never) were mostly individuals without those mental health symptoms. These findings shed light on the extent to which mental health can impact aspects of Hispanic or Latino lived experiences, including but not limited to experiences of discrimination. These results may contribute to understanding the Hispanic or Latino health paradox, which posits that Hispanic or Latino individuals in the US generally have better health outcomes than White individuals, despite lower socioeconomic status [89]. While this paradox may hold true for physical outcomes (using early mortality as the primary health indicator), our findings highlight the need for more research examining the Hispanic or Latino health paradox in the context of mental health. Currently, the paradox is observed most consistently among Mexican individuals [90, 91]. Furthermore, recent studies have found that among Hispanic or Latino individuals, burdens of poor mental health outcomes may be exacerbated by a lower quality of life, and that despite similar prevalences of mental illness [91–94], only about one-third of Hispanic or Latino persons receive mental health treatment compared to nearly one-half of White persons [95]. Thus, future developments in research may benefit from considering the saliency of a Hispanic or Latino health paradox as a function of ethnic origin and mental health outcomes. Nonetheless, individuals with mental health problems may view behaviors through negative biases which can affect their perceptions of discrimination. Some individuals with mental health problems, including depressive symptoms, have cognitive impairments that affect their psychosocial functioning [96]. Future studies, particularly, longitudinal research, should examine the mediating roles of cognitive impairment in the relationship between mental health symptoms and perceived discrimination among Hispanic or Latino individuals.

Furthermore, as recent literature suggests in agreement with our findings, there may be nuances in the health outcomes of foreign-born and US-born Hispanic or Latino individuals that were previously obscured by aggregated data [97]. These findings have concerning implications for not only Hispanic or Latino mental health, but also discrimination against vulnerable individuals within those communities. The findings underscore the importance of understanding and treating mental health symptoms within the Hispanic or Latino population to improve well-being, mitigate exposure to discriminatory experiences, and prevent subsequent cascades of negative health outcomes.

It is also important to acknowledge that certain sociodemographic factors were significantly associated with discrimination irrespective of birthplace, as these findings could inform future research questions around mental health, discrimination, and well-being among the US Hispanic or Latino population. We found that the sociodemographic characteristics of being young, employed, and homeless were associated with higher risks of discrimination among Hispanic or Latino adults. For instance, Hispanic or Latino individuals aged *≥* 50 years were less likely to report discrimination daily/weekly compared to those aged 18–25-years. This finding aligns with a national survey of Hispanic or Latino individuals which found that younger adults were more likely than older adults to report experiences of discrimination [98]. This may be related to mental health trends among young Hispanic or Latino individuals, who are at increased risk for lifetime DSM-IV mood disorders (i.e., depressive and anxiety disorders) compared to White individuals [93, 99]. These mental health symptoms can elevate levels of acculturative stress among both immigrant and non-immigrant Hispanic or Latino young adults [100], who may have fewer accommodative strategies and/or access to resources for coping with such complications compared to their older counterparts [95, 101–103]. These age disparities in mental health may contribute to the increased likelihoods of discrimination among Hispanic or Latino young adults, suggesting the need for understanding the implications of age on perceptions of discrimination and mental health treatment in Hispanic or Latino and other racially and/or ethnically minoritized populations.

The finding that unemployed individuals were less likely to experience discrimination compared to employed individuals may reflect a hostile nature of work environments toward Hispanic or Latino workers, the majority of whom are essential workers [104]. The poor and dangerous work conditions that Hispanic or Latino essential workers are often exposed to [105, 106], coupled with the absence of workplace protections, unsteady employment, and exclusion from certain work benefits can contribute to discrimination and poor health outcomes [106, 107]. Given the context of our mental health findings, it is worth noting that Latino day laborers have been found to experience elevated rates of depression and anxiety [108], which could also potentially explain the increased risk of experiencing discrimination as an employed (vs. unemployed) Hispanic or Latino adult. We further found that homelessness/unstable housing was a significant contributing factor for daily/weekly discrimination. These findings may seem to contradict each other, since unemployment is a well-established cause of homelessness [109]. However, homelessness has been significantly associated with loneliness, which our findings suggest could contribute to heightened risks of discrimination among both foreign-born and US-born Hispanic or Latino individuals [110]. Homelessness is also highly stigmatized, which can heighten risk of discrimination [111]. Mental health symptoms and drug addiction are also common among homeless individuals [112], which can increase their poor treatment or discrimination. This may be especially relevant among young Hispanic or Latino individuals, who have been observed to have higher substance-use rates than other racially and/or ethnically minoritized groups [113]. With the Latino population accounting for nearly a quarter of the US population experiencing homelessness in 2023 [114], there is a need to address all forms of mental health disparity and discriminatory behavior towards this population, who have become especially vulnerable to homelessness/unstable housing since the advent of the COVID-19 pandemic.

Finally, while findings regarding the impact of attribution versus frequency of discrimination remain inconsistent, past research has suggested that attributing discrimination to such specific factors can have increasingly negative personal and interpersonal costs [115]. Given our current study’s observed frequency of reported discrimination and the diversity of reported reasons such as race, gender, and age, further research is needed to explore the role of attributions in the experiences of Hispanic or Latino individuals. This exploratory component of the study raises an especially relevant question regarding perceived discrimination due to immigration status, ancestry or national origin, and race, which were more frequently reported attributions for daily/weekly discrimination among US-born participants than among foreign-born participants. This may present a contradiction or idiosyncrasy in the multiple minority stress theory. Similarly, the most frequent attribution of discrimination to age and height among the overall sample may be associated with economic factors (e.g., employment, productivity), with younger workers and taller individuals less likely to experience economic disadvantages, particularly among immigrants [116–119]. These findings provide additional context in a growing body of research examining discrimination through an intersectional framework [120], and further highlights the need for research to explain potential interpretations for such findings.

Our study has some limitations. The data were derived from a national cross-sectional survey and, therefore, we could not establish causal relationships and/or directionality between mental health symptoms and discrimination—a key direction for future longitudinal research. We therefore reported their associations. In addition, the data collected is self-reported, which presents the potential influence of response bias. It is also important to note that the survey was conducted from May 2021, which was one year after the World Health Organization’s declaration of COVID-19 as a pandemic, and that variables examined within this study are not comprehensive of all factors which might have contributed to discrimination frequency during the pandemic. That is, residual confounding variables (e.g., English proficiency, acculturation or length of stay in the US, mental health disorders’ impacts on cognitive internalization of discrimination) may be present in this study. Moreover, while the survey was nationally distributed, there’s a possibility that responsiveness and survey completeness varied by factors such as acculturation and region, though this information would be difficult to discern given the proprietary nature of the survey recruitment process by Qualtrics. This highlights a need for oversampling underrepresented Hispanic or Latino ethnic subgroups to more holistically grasp important within- and between-group differences. Thus, the small cell sizes or counts in some groups (e.g., larger US-born/non-immigrants) could have resulted in the larger confidence intervals in those groups, as sample sizes influence confidence intervals [121]. Furthermore, the survey being administered online and in English could have also affected participation based on English literacy and access to technology, which may have also varied across ethnic subgroups due to factors such as acculturation. Additionally, this study did not include the source of discrimination. Individuals living with mental health issues may experience discrimination from their own communities [88, 122, 123], therefore this should be considered in future studies.

## Conclusion

Using a national survey of US Hispanic or Latino adults, this study found that during the COVID-19 pandemic, both US-born and foreign-born individuals experienced high rates of discrimination. However, non-immigrant individuals reported higher prevalences than their immigrant counterparts. While Hispanic or Latino persons in general were significantly likely to experience discrimination if they experienced loneliness, non-immigrant persons alone were significantly likely to experience discrimination if they also experienced anxiety/depression. In the context of birthplace and mental health symptoms, our study contributes to a more comprehensive and multi-dimensional perspective of Hispanic or Latino experiences and needs in the US. While it has been recognized that the Hispanic or Latino population may experience higher rates of mental health symptoms compared to other racial and/or ethnic groups [93, 99], very little research has investigated mental health as a potential risk factor for discrimination, especially while stratifying by birthplace.

The findings of this study are especially relevant given that the Hispanic or Latino population is prone to underutilization of mental health treatment [95, 124]. Given the nature of our findings, along with recent findings elucidating neural mechanisms that may link mental health to perceived discrimination [79, 80], it is crucial that future research questions and hypotheses consider additional factors which may impact experiences of mental health and discrimination among Hispanic or Latino individuals. Finally, since the advent of the pandemic, there has been limited research exploring attributions for discrimination stratified by birthplace. Our findings underscore a need for a more thorough investigation of reasons for discrimination as a factor of birthplace.

The Hispanic or Latino population is not a monolith, and individuals’ experiences can vary by not only birthplace but also factors such as country of birth, acculturation, and generational status. By adopting research practices and scientific inquiry that thoroughly and holistically examine the unique experiences and exposures of Hispanic or Latino individuals, future public health initiatives can effectively address the specific needs within this population, as well as inform the public about the characteristics and consequences of perpetuating discrimination. Thus, such developments can improve population health and take more meaningful steps toward mitigating discrimination and achieving health equity.

## Electronic supplementary material

Below is the link to the electronic supplementary material.

Supplementary Material 1

## Data Availability

The data are available by making a request through Dr. FW per the new Data Management and Sharing Agreement plan.

## Abbreviations

US: United States
IRB: Institutional Review Board
NIH: National Institutes of Health
GAD-2: 2-item Generalized Anxiety Disorder
PHQ-2: 2-item Patient Health Questionnaire
PHQ-4: 4-item Patient Health Questionnaire
RRR: Reported relative risk ratio
CI: Confidence interval
