# Supplementary material for: Mental health symptoms and discrimination among immigrant and US-born Hispanic or Latino adults during the COVID-19 pandemic
Source: BMC Psychol. 2025 May 13;13:504. doi: 10.1186/s40359-025-02798-7 (PMC12076878; doi:10.1186/s40359-025-02798-7)
Supplement: Supplementary file 1 — Supplementary Material 1 [file 40359_2025_2798_MOESM1_ESM.docx]

**Supplemental Online Material**

**Mental health symptoms and discrimination among immigrant and US-born Hispanic or Latino adults during the COVID-19 pandemic**

**Authors**

Emmanuel A. Odame^1#^, Maryam Elhabashy^2#^, David Adzrago^2^, Jolyna Chiangong^2^, Cameron K. Ormiston^2,3^, Faustine Williams^2^

#Equally contributed as joint first authors

**eTable 1.1.** Polytomous logistic regression analysis of the associations between anxiety, depression, loneliness, and place of birth related with reports of discrimination frequency among Hispanics or Latinos (n= 1,126).

|  | **Discrimination frequency (Reference Category: Never)** | | | | |
| --- | --- | --- | --- | --- | --- |
|  | **Daily or at least once a week** | |  | **About once a month** | |
|  | **RRR** | **95% CI** |  | **RRR** | **95% CI** |
| **Mental health symptoms** |  |  |  |  |  |
| Anxiety | 1.14 | (1.00, 1.29) |  | 1.05 | (0.80, 1.23) |
| Depression | 1.21** | (1.06, 1.39) |  | 0.94 | (0.79, 1.11) |
| Loneliness score | 1.30*** | (1.18, 1.44) |  | 1.34*** | (1.20, 1.50) |
| **Place of birth** |  |  |  |  |  |
| Foreign-born (Immigrant) | 0.67* | (0.49, 0.93) |  | 0.67* | (0.46, 0.97) |
| US-born (Non-immigrant) | Ref |  |  | Ref |  |

*RRR = Relative risk ratio. 95% CI = 95% confidence interval. Statistical significance at *p<0.05, **p<0.01, and ***p<0.001. Ref= reference.
Adjusted for sociodemographic factors (age, gender identity, sexual orientation, marital status, education, annual household income, employment status, and homelessness/unstable housing) in polytomous logistic regression model.*

**eTable 1.2.** Polytomous logistic regression analysis of the associations between anxiety/depression, loneliness, and place of birth related with reports of discrimination frequency among Hispanics or Latinos (n= 1,126).

|  | **Discrimination frequency (Reference Category: Never)** | | | | |
| --- | --- | --- | --- | --- | --- |
|  | **Daily or at least once a week** | |  | **About once a month** | |
|  | **RRR** | **95% CI** |  | **RRR** | **95% CI** |
| **Mental health symptoms** |  |  |  |  |  |
| Anxiety/depression | 1.17*** | (1.11, 1.24) |  | 1.00 | (0.93, 1.06) |
| Loneliness score | 1.30*** | (1.18, 1.44) |  | 1.34*** | (1.19, 1.50) |
| **Place of birth** |  |  |  |  |  |
| Foreign-born (Immigrant) | 0.68* | (0.49, 0.93) |  | 0.66* | (0.46, 0.96) |
| US-born (Non-immigrant) | Ref |  |  | Ref |  |

*RRR = Relative risk ratio. 95% CI = 95% confidence interval. Statistical significance at *p<0.05, **p<0.01, and ***p<0.001. Ref= reference.
Adjusted for sociodemographic factors (age, gender identity, sexual orientation, marital status, education, annual household income, employment status, and homelessness/unstable housing) in polytomous logistic regression model.*

**eTable 2.1.** Polytomous logistic regression analysis of the associations between anxiety, depression, and reports of discrimination among foreign-born and US-born Hispanics or Latinos.

|  | **Foreign-born (Immigrant)** | |  | **US-born (Non-immigrant)** | |
| --- | --- | --- | --- | --- | --- |
|  | **Base/Reference Category: Never** | |  | **Base/Reference Category: Never** | |
|  | **Daily or at least once a week** | **About once a month** |  | **Daily or at least once a week** | **About once a month** |
|  | **RRR (95% CI)** | **RRR (95% CI)** |  | **RRR (95% CI)** | **RRR (95% CI)** |
| **Mental health symptoms** |  |  | |  |  |
| Anxiety | 1.13 (0.93, 1.38) | 0.97 (0.75, 1.25) | | 1.15 (0.0.97, 1.38) | 1.09 (0.89, 1.34) |
| Depression | 1.16 (0.94, 1.43) | 1.02 (0.78, 1.33) | | 1.28** (1.07, 1.54) | 0.91 (0.73, 1.13) |
| Loneliness score | 1.28** (1.10, 1.48) | 1.35** (1.14, 1.60) | | 1.34*** (1.16, 1.53) | 1.37*** (1.17, 1.61) |

*RRR = Relative risk ratio. 95% CI = 95% confidence interval. Statistical significance at *p<0.05, **p<0.01, and ***p<0.001. Ref= reference.*

*Adjusted for sociodemographic factors (age, gender identity, sexual orientation, marital status, education, annual household income, employment status, and homelessness/unstable housing) in polytomous logistic regression model.*

**eTable 2.2.** Polytomous logistic regression analysis of the associations between anxiety/depression and reports of discrimination among foreign-born and US-born Hispanics or Latinos.

|  | **Foreign-born (Immigrant)** | |  | **US-born (Non-immigrant)** | |
| --- | --- | --- | --- | --- | --- |
|  | **Base/Reference Category: Never** | |  | **Base/Reference Category: Never** | |
|  | **Daily or at least once a week** | **About once a month** |  | **Daily or at least once a week** | **About once a month** |
|  | **RRR (95% CI)** | **RRR (95% CI)** |  | **RRR (95% CI)** | **RRR (95% CI)** |
| **Mental health symptoms** |  |  | |  |  |
| Anxiety/depression | 1.14** (1.05, 1.24) | 0.99 (0.89, 1.10) | | 1.21*** (1.12, 1.30) | 1.00 (0.91, 1.10) |
| Loneliness score | 1.28** (1.10, 1.48) | 1.35** (1.14, 1.60) | | 1.33*** (1.16, 1.53) | 1.37*** (1.17, 1.61) |

*RRR = Relative risk ratio. 95% CI = 95% confidence interval. Statistical significance at *p<0.05, **p<0.01, and ***p<0.001. Ref= reference.*

*Adjusted for sociodemographic factors (age, gender identity, sexual orientation, marital status, education, annual household income, employment status, and homelessness/unstable housing) in polytomous logistic regression model.*
